# Comparison of Five Presbyopia-Correcting Intraocular Lenses: Optical-Bench Assessment with Visual-Quality Simulation

**DOI:** 10.3390/jcm12072523

**Published:** 2023-03-27

**Authors:** Grzegorz Łabuz, Weijia Yan, Isabella D. Baur, Ramin Khoramnia, Gerd U. Auffarth

**Affiliations:** Department of Ophthalmology, David J. Apple International Laboratory for Ocular Pathology, Heidelberg University, 69120 Heidelberg, Germany

**Keywords:** trifocal intraocular lens, optical quality, modulation-transfer-function

## Abstract

Presbyopia correction through implantation of a trifocal intraocular lens (IOL) is a modality offered to both cataract and refractive-lens exchange patients. To maximize postoperative satisfaction, IOL selection needs to be made based on patients’ requirements aligned with the available technology. Five Trifocal IOLs were assessed in this study, and their differentiating features were identified: Triumf POD L GF, AT Lisa Tri, Tecnis Synergy, AcrySof IQ PanOptix, and Acriva Trinova Pro C. The optical quality was assessed using the modulation-transfer-function principle. Simulated defocus curves were derived from a non-linear formula. Far-focus simulated visual acuity (simVA) was 0.03 logMAR or better for all the studied IOLs, showing minimal differences. However, each IOL’s intermediate focus position differed across a range from 61 cm to 80 cm; and for the near focus, it was 36 cm to 44 cm. Triumf demonstrated improved intermediate point at the expense of the near focus resulting in a lower predicted near VA. PanOptix exhibited the shortest range of vision without a clear distinction between intermediate and near-point. The remaining lenses presented three foci of comparable optical quality and, thus, simVA performance. Each model, however, revealed a different aperture-change response. Trinova function improved at intermediate but was worse at near for larger pupils. The opposite was observed for AT Lisa. Synergy’s optical quality change was predominantly associated with lower pupil diameter. In conclusion, the trifocal IOLs can be differentiated according to their secondary-foci position, light-energy distribution, and pupil-size-related behavior. The observed differences may translate directly into a clinical effect showing that the trifocal IOLs vary in their ability to deliver optimal vision at different distances, with some providing improved intermediate while others favor reading distance. The knowledge gained through this objective testing can support IOL selection, postoperative patient counselling and increase the chance of spectacle independence after surgery.

## 1. Introduction

In routine crystalline-lens extraction and monofocal intraocular lens (IOL) implantation, the loss of visual quality at a range of distances is attributed to the inability of the artificial lens to accommodate. A trend of lowering the age of patients scheduled for cataract surgery whose occupational and leisure activities require good vision at various distances creates the need for presbyopia-correcting lenses [1]. Non-cataract patients with accommodative deficiency wishing to undergo refractive lens exchange with the objective of spectacle independence constitute another group, and they typically have high expectations of the postoperative outcome [2]. Recent developments in trifocal technology have led to the success of these surgical procedures [3].

When choosing a trifocal IOL to fully meet the patient’s requirements, the diversity of trifocal lenses available, as well as the often-incomplete information provided by the lens manufacturer, can make lens selection difficult. Measuring and assessing IOLs at an independent research laboratory may address this issue by objectively comparing technology under controlled conditions. Recent translational studies demonstrated that optical-quality metrics of presbyopia-correcting IOLs correlate highly with postoperative vision outcomes, indicating a close correspondence between the optical design and the patient’s functional result [4,5]. Although this approach is only limited to estimating the average eye effect, it may predict clinically relevant differences between IOLs. A recent study by Yan et al. demonstrated a close correspondence between a laboratory-derived defocus curve and a clinical outcome; however, in that study, the impact of applied monochromatic and chromatic aberrations was not accounted for, despite being recognized as an important factor affecting the eye’s imaging performance and the depth of focus [6,7]. In addition, the latest introduction of novel trifocal technology requires continuous research, which may support preoperative decision-making and manage expectations.

Since the introduction of the concept of trifocality [8], various designs have been proposed. The literature shows that designs featuring several Fresnel rings can be considered the leading approach [3,8,9,10,11,12,13]. Despite similarities in the working principle, such as light diffraction, each diffractive-refractive lens has its own characteristic. Besides the location of the intermediate and near points, which is set by the diffractive-ring spacing, trifocal lenses also differ in their light energy distribution modulated by the height of the steps. Another aspect influencing the energy allocation is the pupil size, either because of the cessation of the diffractive structure at larger apertures, or peripheral modification of the lens design to reinforce one or two selected foci. Besides differences in the correction of corneal spherical aberration (SA), current lens designs also intend to alter the eye’s chromatic aberration [6,9,14], the effects of which are challenging to gauge clinically. In the laboratory, however, the impact of both aberrations can be directly evaluated by changing the measurement conditions.

The optical quality of modern trifocal IOLs was assessed under monochromatic and polychromatic conditions, as well as with an aberration-neutral corneal model and one representing the average SA level reported in population studies [15]. Preclinical visual quality metrics were derived from the measured optical quality and compared. Furthermore, the impact of aperture size on the optical performance was assessed to study the IOLs’ pupil dependency and provide additional information on the existing and recently-introduced trifocal technology.

## 2. Materials and Methods

### 2.1. Intraocular Lenses

Five trifocal IOLs were evaluated: FineVision Triumf POD L GF (BVI Medical, Liege, Belgium), AT Lisa Tri 839 MP (Carl Zeiss Meditec AG, Berlin, Germany), Tecnis Synergy (Johnson & Johnson, Santa Ana, CA, USA), AcrySof IQ PanOptix (Alcon, Fort Worth, TX, USA), and Acriva Trinova Pro C Pupil Adaptive (VSY Biotechnology, Istanbul, Turkey). Two samples per model were analyzed, each with +20 D power.

FineVision Triumf POD L GF is a hydrophobic acrylic IOL with a 1.53 refractive index and a 42 Abbe number [16]. The biconvex optic incorporates a proprietary chromatic aberration-correcting technology to enhance polychromatic lens performance. It features a trifocal full-diffractive 6 mm non-apodized design, rendering pupil-independent light-energy allocation, with the intermediate focus at 1.75D and the near focus at 3.50 D. According to the manufacturer, the IOL elongates the depth of focus by a unique energy split, which favors intermediate over near distance, and allocates approximately 50% of light to far, 30% to intermediate, and 20% to near. The base lens of the Triumf has an aspheric profile correcting −0.11 μm of positive SA of the eye.

The AT Lisa Tri 839 MP is a (25%) hydrophilic acrylic IOL with a hydrophobic surface. The lens has a refractive index of 1.46 and an Abbe number of 56.5 [17]. A full-diffractive non-apodized design yields an intermediate focus at 1.66 D and a near focus at 3.33 D. An asphericity of −0.18 μm compensates corneal SA. AT Lisa’s energy distribution is (approx.) 48% at far, 31% at near, and 21% at intermediate for a 3 mm pupil. However, this light-split is aperture dependent as the lens turns bifocal above a 4.34 mm diameter.

The Tecnis Synergy is a hydrophobic acrylic IOL. It has a refractive index of 1.47 and an Abbe number of 55. The IOL’s front surface is aspheric, and chromatic aberration is compensated through proprietary technology, of which the effectiveness has yet to be verified. Like other Tecnis IOL models, the Synergy maintains −0.27 μm of SA correction. The manufacturer provides no information on the add-power position or the energy allocation.

The IQ PanOptix is composed of a hydrophobic AcrySof material. Its refractive index is 1.55, and it has increased dispersion due to its lower Abbe number, which equals 37 [18,19]. PanOptix’s design lowers corneal SA with −0.10 μm asphericity. Although the PanOptix is a non-apodized lens, the diffractive pattern covers a 4.5 mm lens area, with the outer part being monofocal. The IOL’s trifocal optics attributes 50% of light to the far-point, whereas intermediate (2.17 D) and near (3.25 D) foci received 25% each.

The Acriva Trinova Pro C Pupil Adaptive is made of hydrophilic acrylic material with a refractive index of 1.46 and an Abbe number of 58 [20]. The IOL adds +3.60 D for near and +1.80 D for intermediate vision through a sinusoidal-diffractive profile [21]. According to the manufacturer, the smoothed and progressively-changing diffractive profile reduces scattered light and optimizes light distribution. The IOL yields far-dominant performance and uneven light energy distribution to the other foci and allocates about 21% for intermediate and 36% for near at 3 mm. Given, however, its apodized design, referred to as Pupil Adaptive technology, these proportions change to an equal 28% split between the two foci at 4.5 mm. Trinova’s asphericity yields −0.10 µm of SA correction.

### 2.2. Metrology Setup

The assessment was performed using an OptiSpheric IOL PRO2 (Trioptics GmbH, Wedel, Germany) device. It consists of a polychromatic illumination system composed of a collimator and an LED light source with a broad spectrum allowing for the selection of various spectral conditions. The setup features a range of apertures and corneal models placed on rotation wheels. An IOL under test is placed in a mechanical holder with flat windows and submerged in a balanced salt solution. An acquisition module that utilizes a set of objective lenses and a monochromatic camera (VA−1MCM120-A0-C; Vision Systems Technology, Vista, CA, USA) was used for image recording. OptiSpheric IOL PRO2 is ISO-11979 compliant and has a high accuracy of 2% for optical-quality measurements with an acceptable error of power measurements of less than 0.3% [6,10,22]. In this study, the trifocal IOLs were compared using standard settings featuring a monochromatic filter (546 nm) and an aberration-neutral corneal model (achromatic doublet, f = 36 mm), referred to as Condition 1. However, given that such an arrangement may poorly represent an in vivo lens performance, a polychromatic (V-Lambda) filter and a model cornea (singlet lens, f = 39 mm) with +0.27 µm of SA and 1 D of chromatic aberration [22] were introduced, denoted as Condition 2.

### 2.3. Optical-Quality Metrics

The impact of each condition on the optical quality was evaluated based on the modulation transfer function (MTF) derived from a crosshair target in sagittal and tangential meridians. Since all studied IOLs are rotationally symmetric, MTF values along the two perpendicular meridians were averaged. The measurements were conducted at three primary foci, i.e., far, intermediate, and near, and for 3- and 4.5-mm pupils. The defocus tolerance of each IOL was evaluated at 3 mm using the area under the MTF up to 50 lp/mm (equivalent to 15 cyc/deg), referred to as MTFa [4]. The through-focus MTFa was measured over a +1 to −3.5 D range at the spectacle plane, corresponding to +1.33 and −4.67 D at the IOL plane. Note that add powers provided by manufacturers always refer to the IOL plane. Simulated visual acuity (SimVA) was calculated using the method devised by Alarcon et al. [4]. The through-focus MTF at 50 lp/mm was measured at 2, 3, 4, and 5 mm to study the IOLs’ pupil dependency.

## 3. Results

### Optical Quality Assessment

**Figure 1:**
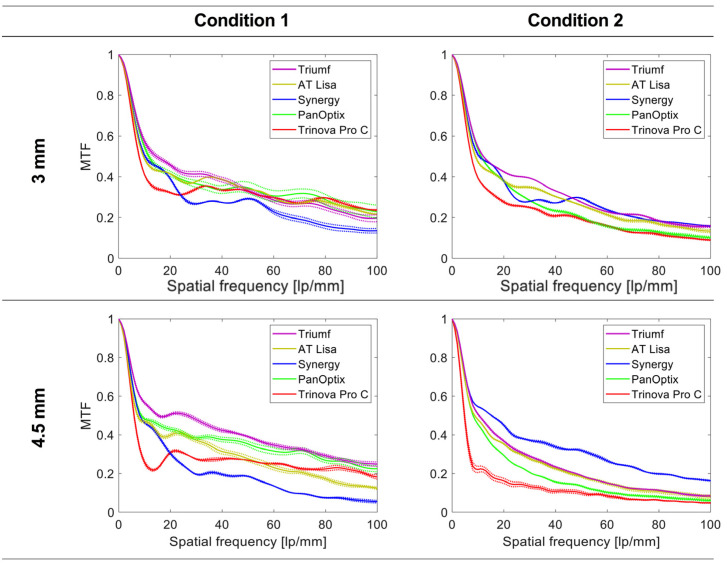
Modulation transfer function (MTF) levels up to 100 lp/mm (equivalent to 30 cyc/deg) of the trifocal intraocular lenses at the far focus for 3- and 4.5-mm pupils. Monochromatic light with an aberration-neutral corneal model was applied in Condition 1, and polychromatic light with a population level of corneal spherical aberration in Condition 2. The dotted lines show the values of each lens separately; the solid lines refer to the average of two samples.

Figure 1 shows far-point MTF curves of the studied IOLs for the 3- and 4.5-mm aperture and the two conditions. At 3 mm, a monochromatic MTF of the Trinova was lower than at spatial frequencies below 24 lp/mm, but above this level, its optical quality was within the range of the other trifocals. The Synergy demonstrated lower MTF values for higher-spatial frequencies than the other lenses. The three remaining trifocals showed overall comparable performance. In Condition 2, Trinova’s and PanOptix’s MTF was lower than that of the other IOLs at 3 mm. AT Lisa’s far performance was better but still reduced in polychromatic light. However, the two chromatic-aberration correcting lenses were only minimally affected by the change in the spectral conditions.

At 4.5 mm, the Trinova and the Synergy demonstrated a similar behavior as at the lower aperture in Condition 1. The Triumf showed the highest monochromatic MTF levels at no SA, followed by the PanOptix and the AT Lisa. In Condition 2, however, the Synergy demonstrated improved optical quality, with the Trinova showing the most reduced values.

**Figure 2:**
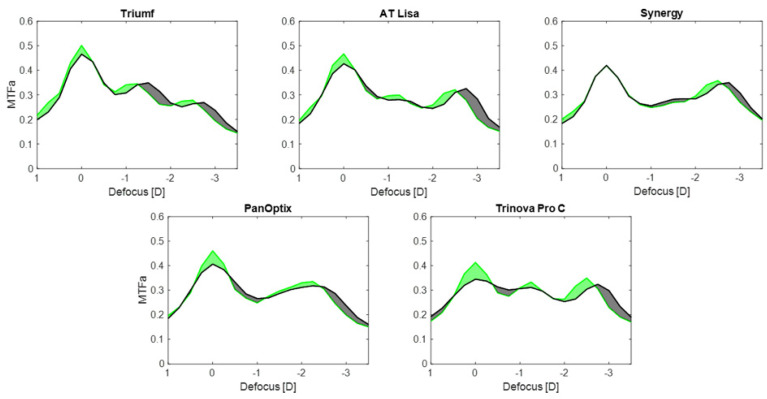
The area under the modulation transfer function (MTFa) of the studied IOLs: from measurements obtained at 3 mm and a defocus range from +1 D to −3.5 D. Monochromatic light with an aberration-neutral corneal model was applied in Condition 1 (green lines), and polychromatic light with a population level of corneal spherical aberration in Condition 2 (black lines). Green-shaded areas indicate an improved MTFa in Condition 1 over Condition 2, and black-shaded areas denote a higher optical quality in Condition 2.

Figure 2 reports the MTFa change for the five IOLs under the studied conditions. The Triumf exhibited a 7% loss of the MTFa in polychromatic light compared to the monochromatic one. The heights of the secondary peaks were shifted by 0.25 D but comparable, which was close to MTFa = 0.35 at intermediate and MTFa = 0.27 at near. AT Lisa’s loss at far was minimally higher—9%—with a slight intermediate-MTFa difference of 0.03 in favor of Condition 1. At near, the MTFa was almost identical, and about MTFa = 0.32. For the Synergy, no difference was observed between monochromatic and polychromatic conditions at far focus. The intermediate range was improved in Condition 1 by only MTFa = 0.01, with the same difference affecting the near comparison. The change from Conditions 1 to 2 resulted in an MTFa reduction of 11% in the PanOptix. Its intermediate and near foci merged, showing a minimally higher near-MTFa peak by 0.02 in monochromatic than in polychromatic conditions. The Trinova showed an MTF loss of 17% at the far focus and slight differences at the intermediate (by MTFa = 0.02) and near (by MTFa = 0.03) points.

Figure 2 also indicates that the application of Condition 2 extended the depth of focus, particularly improving the optical quality between the far and the intermediate focus. A near-focus shift can also be observed in the MTFa results.

**Figure 3:**
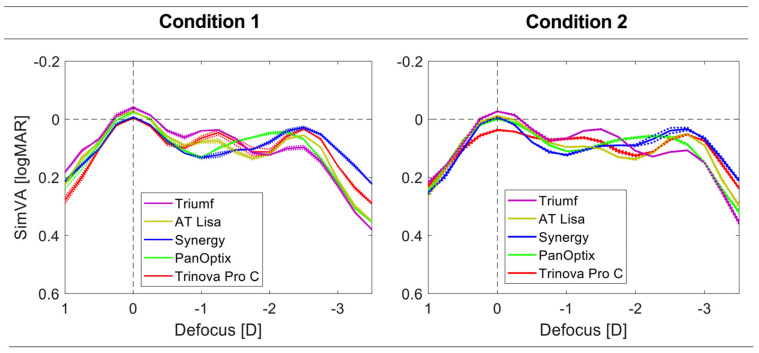
The simulated visual acuity (SimVA) of the trifocal IOLs was derived from optical assessment at 3 mm under two conditions: Condition 1 had the lenses tested in monochromatic light with an aberration-neutral corneal model. Condition 2 used polychromatic light which had a population level of corneal spherical aberration. The dotted lines show the values of each lens separately; the solid lines refer to the average of two samples.

Visual acuity simulations from the optical measurements are presented in Figure 3. In Condition 1, all IOL models reached 0.00 logMAR or better, but it was not the case for Trinova in Condition 2 (logMAR = 0.03). The Triumf demonstrated the highest far- and intermediate-VA for both conditions, but the expected VA at near was lower than that of the other trifocals. Trinova’s and Triumf’s intermediate VA was close, but the former demonstrated a better outcome at near. Although the strongest VA peak at near was detected with the Synergy, it was closely followed by the Trinova and the AT Lisa. The PanOptix did not reveal a transition interval between the two foci, showing about a +0.25 D difference in the reading distance between the other four lenses. A similar shift can be observed between the secondary-foci position between Conditions 1 and 2, with the latter providing a more extended depth of focus by approx. +0.25 D.

**Figure 4:**
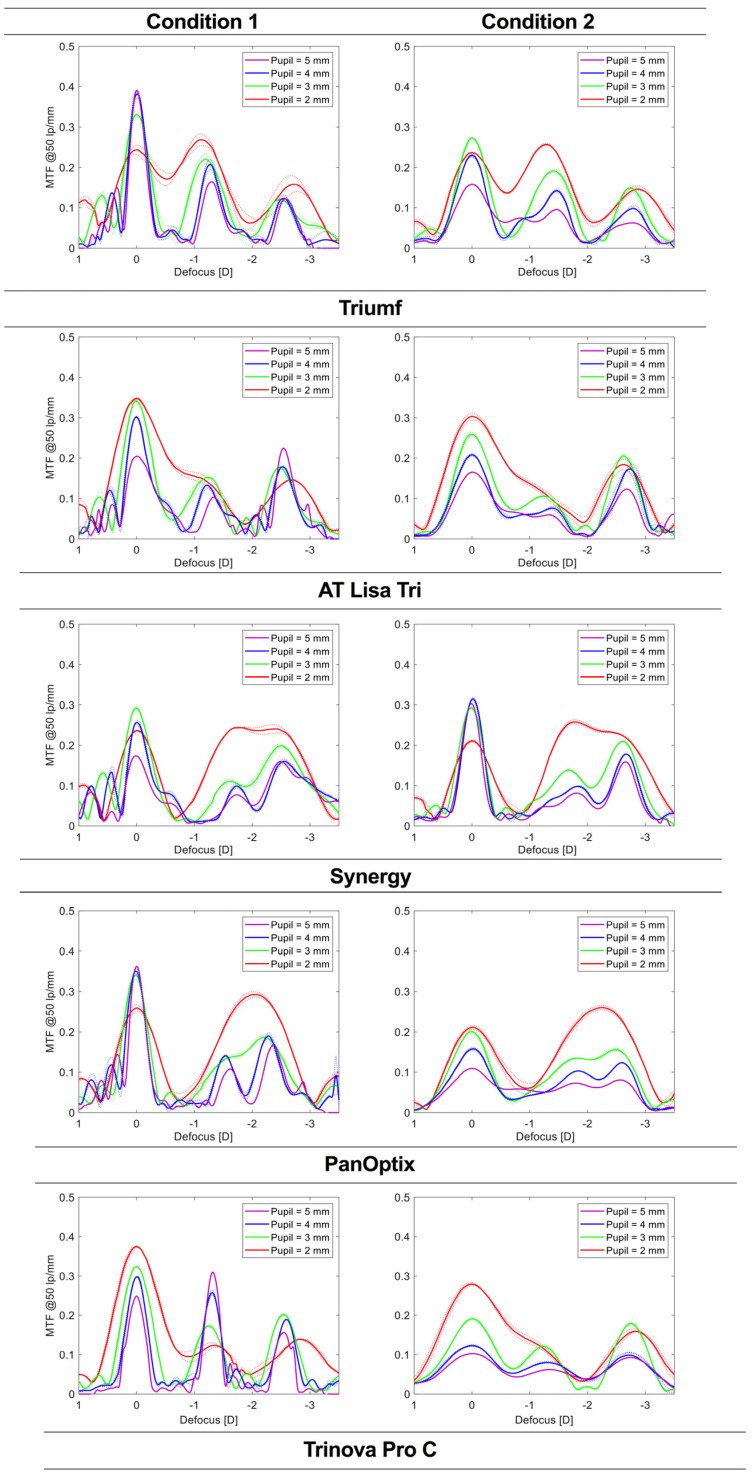
The through-focus modulation transfer function (MTF) curves at 50 lp/mm (equivalent to 15 cyc/dec) and at various pupil sizes. Monochromatic light with an aberration-neutral corneal model was applied in Condition 1, and polychromatic light with a population level of corneal spherical aberration in Condition 2. The dotted lines show the values of each lens separately; the solid lines refer to the average of two samples.

The results of the through-focus modulation transfer function (TF MTF) measurements at a frequency of 50 lp/mm presented in Figure 4 demonstrate how pupil size may affect the behavior of the trifocal IOLs. At 2 mm, the TF analysis revealed the merging of two foci, which for the Triumf, AT Lisa, and Trinova (only Condition 2) affected the far-intermediate distance, but for the Synergy and the PanOptix, the intermediate-near distance. Far-focus MTF was improved for the AT Lisa and the Trinova, but it weakened for the remaining IOLs. At 3 mm, all IOLs exhibited a trifocal behavior—three peaks corresponding to design foci. Still, PanOptix’s intermediate focus was only slightly marked. The Trinova demonstrated the largest pupil-dependency with higher MTF values at the intermediate than at the near focus when the aperture was increased to 4 mm (Condition 1). In Condition 2, however, Trinvoa’s foci are less distinct and have close MTF values. Otherwise, no major differences were observed between Conditions 1 and 2. At 5 mm, the Trinova demonstrated an intermediate focus dominance, while AT Lisa’s MTF was the highest at the near range. The PanOptix and the Triumf showed a good optical performance at far in Condition 1, but for the Synergy, the same conclusion can be made on its far MTF in Condition 2.

## 4. Discussion

In this study, the comparison of contemporary trifocal-IOL technology was performed, providing an objective measure of the optical quality and the simulated clinical effect. The presented analysis highlighted the differences between the lenses, mainly related to the location of secondary foci, light energy distribution, pupil dependency, and the management of dispersion effects and SA. Although using monochromatic conditions yielded a higher imaging quality, the introduction of chromatic aberration resulted in an increased depth of focus.

The add power of trifocal lenses is one of the main factors differentiating the studied IOLs. Despite differences between the exact location of the intermediate and near foci, the clinical differences are minimized by the resolution of testing, which was assessed in vitro with a 0.25 D step. But in clinical evaluation, typically, a 0.50 D increment is used, which may pose a challenge in precisely detecting IOLs’ design foci. The Triumf, the AT Lisa, and the Trinova exhibited the intermediate VA peak at 80 cm, which contrasts with the 67 cm peak location of the Synergy detected in the current assessment. Although Synergy’s manufacturer does not provide the intermediate-focus specification, its position could have been expected, given that this IOL was designed based on a Tecnis Symfony, which shows a similar outcome [11]. A recent evaluation of refractive-lens-exchange patients implanted with the Synergy confirms these laboratory findings with a preferred interme-diate and near distance of 72.4 ± 6.4 cm and 36.9 ± 3.0 cm, respectively, reported by Baur et al. [23]. The intermediate range of the PanOptix appears closer to the eye, about 61 cm. Still, it was not distinguishable in the VA simulations, and only at 4 mm, its presence was clearly marked in the TF MTF analysis. Therefore, it appears that no consensus has been found on the position of the intermediate focus, which ranges from 61 cm to 80 cm. By contrast, the near-focus position seems to be minimally different in all the studied IOLs but one. PanOptix’s near focus, seen as the secondary peak in Figure 2 and Figure 3 and tertiary peak in Figure 4, was farther away than the other IOLs, irrespective of the condition used. Understanding the differences between the offered intermediate vision may improve patient selection depending on the patient’s needs. This information should also be considered in postoperative assessment to gauge the effectiveness of an implanted trifocal lens.

Besides the add-power design, the light energy split between the three foci is also important. This aspect may explain the difference in the performance of the Triumf and the other IOLs. Since this lens favored intermediate rather than near vision, its optical quality and expected VA were better at the former position. Kim et al. compared the visual outcomes of a mix-and-match procedure involving the Triumf and a standard trifocal lens (FineVision HP) of the same manufacturer, and an EDoF AT Lara with an AT Lisa Tri (both from Carl Zeiss) [12]. They reported a monocular defocus curve of the Triumf IOL implanted in 106 subjects. Although Kim’s group confirmed the expected VA decline with defocus, the intermediate focus position appeared flattened without a clear secondary peak. The clinical best-corrected VA obtained monocularly was approximately 0.01 logMAR, which contrasts with −0.04 logMAR predicted (binocularly), indicating the contribution of non-optical factors. Still, the predicted reduction in VA at near compared to intermediate distance was reflected by Kim et al.’s assessment [12]. They found a logMAR VA of (approx.) 0.18 at −1.50 D, which was reduced to 0.23 at −2.50 D. By comparison, in the current evaluation, a 0.08 logMAR difference in Condition 2 was noted. In that clinical study, the Triumf and the FineVision HP IOL differed in VA at −2.50 D by 0.12 logMAR in favor of the standard trifocal. However, no differences between these two IOLs were observed at −1.50 D. In the present study, the VA gap between the Triumf and the other IOLs ranged from 0.05 to 0.08 logMAR. More clinical data are needed to verify those findings and to refine the VA-prediction formula for the Triumf IOL.

The light-energy partition is closely related to the optical quality of multifocal IOLs, as demonstrated through a ray-propagation study [18]. According to the manufacturer, the Trinova Pro C has a distinctive light distribution with more energy diverting to near than intermediate focus at 3 mm and an even split at 4.5 mm. The TF MTF measurements confirmed this property in Condition 1, which is the most representative of the design features due to the lack of confounding effect of chromatic aberration and minimizing SA in this lens. In Figure 4, a nearly equal MTF at 50 lp/mm was found at the far and intermediate point of the Trinova at 4 mm. At a higher aperture, an intermediate dominance was reported in a different study, which is also seen in the current results at 5 mm [21]. Nevertheless, the concept may be particularly advantageous in a situation like driving, to prioritize focusing on the road and the dashboard while disregarding the near range. Also, a gradual disappearance of the third focus may potentially also lower the perception of photic phenomena [24]. Though, this conjecture has to be verified.

The TF MTF also demonstrated the design features of the AT Lisa, which turns bifocal above the 4.34 mm diameter. By contrast to the Trinova, the AT Lisa favors the near focus, which, in Condition 1, shows a higher discrete MTF value than that at no defocus. The PanOptix, however, introduced a different approach with a cessation of the diffractive structure above 4.5 mm. Similarly, the Synergy shows a refractive periphery with the last diffractive ring with a diameter of about 5 mm. As a result, the contribution of the secondary foci diminishes with a simultaneous improvement of the far focus. Despite those measures to enhance or diminish the weight of specific foci when the pupil diameter increases, the contribution of the central power still effectively creates three distinct points seen in the TF MTF measurements. Thus, their contribution to unwanted-light phenomena cannot be excluded.

Figure 4 demonstrates that SA degrades the imaging performance of IOLs at 4- and 5-mm apertures, which is consistent with the findings from the literature [25]. The magnitude of the quality loss depends on the selected conditions. For instance, if the aberration-neutral corneal model is used, which is not representative of the human cornea [15], the Synergy lens shows over a 40% MTF reduction at 50 lp/mm of the far point compared to Condition 2 measurements. This can be understood as the Tecnis asphericity yields −0.27 µm of primary SA; hence, Condition 1 appears particularly unfavorable for the Synergy and other models featuring a higher level of SA compensation. The Triumf demonstrated the opposite behavior; however, in this case, besides SA, which is reduced by 0.11 µm in Condition 2, chromatic-aberration effects also play a confounding role [14]. Comparable conclusions can be drawn from the optical performance of the PanOptix and the Trinova, given their close SA correction (−0.10 µm). AT Lisa’s SA correction lies nearly in the middle (−0.18 µm) of the values implemented in the other trifocals; therefore, leaving some of the corneal SA uncorrected appears to be the dominant approach. Laboratory studies have demonstrated that an aspheric lens with a full corneal SA compensation in well-centered conditions may produce a superior image quality over a spherical or aberration-neutral lens [26]. Still, the range of SA values found in the cataract population and IOL misalignment observed after uncomplicated implantations make this situation difficult to replicate in vivo [15]. This may explain why some clinical studies have failed to demonstrate the benefits of SA-correcting lenses, with only some showing better mesopic contrast sensitivity but without improving VA [27]. A low-aspheric design has proven more robust against IOL’s tilt and decentration [28]. However, moderate decentration 0.27 mm and tilt 2.89° are also well tolerated in patients implanted with a Tecnis IOL despite a higher SA correction [29]; but, the presence of a positive SA of the cornea may be beneficial for a pseudophakic eye due to its effect on the depth of focus [30]. Therefore, it is still a subject of debate which of the proposed asphericities would be the most advantageous in terms of the optical quality and the depth of focus; or perhaps, a customized approach can be developed that will mitigate the impact of the eye’s imperfections more effectively [31].

Similar to SA, uncorrected chromatic aberration may also have the advantage of extending the range of vision [32]. In Figure 2, the gray area extending between the far and intermediate focus and beyond the near focus indicates improved optical quality. This improvement may translate into patients experiencing a better tolerance to defocus in po-lychromatic light. The transition from monochromatic to polychromatic condition yielded a near-point shift by +0.25 D, indicating the gain in the range of vision. Given the applied aperture size (i.e., 3 mm) and the low SA levels expected, the effect appears to stem from chromatic aberration present only in Condition 2. However, one cannot disregard the contribution of the apparent differences between the two corneal models in the optical setup. The aberration-neutral version consists of a doublet with a 36 mm focal length, but the aberrated lens is a single with a focal length equal to 39 mm. Although small, this difference may also affect the scaling of the diopter range. Nevertheless, the application of chromatic aberration to extend the depth of focus has already been researched extensively [32,33], and our results further indicate that ocular dispersion may improve this aspect. On the other hand, the introduction of polychromatic light resulted in reduced optical performance, particularly noticeable at Trinova’s and PanOptix’s far-point. The clinical impact of the observed changes has yet to be elucidated since the human eye’s susceptibility to light dispersion appears to be low [32]. Furthermore, the interaction of chromatic and monochromatic aberrations has been shown to provide additional visual benefits [34], which should also be considered when designing an IOL. More research is needed on the effects of chromatic aberration on the depth of focus and the visual quality of pseudophakia.

## 5. Conclusions

In conclusion, modern trifocal IOLs differ in their add powers, which may provide optimal vision between 61 cm and 80 cm for intermediate, and 36 cm and 44 cm for reading, depending on the IOL and the measurement conditions that are used (Figure 2 and Figure 3). The results on the best-focus position, seen as a simVA peak, may be applied in the IOL-selection process depending on the patient’s preferred distance, which could increase the chance of spectacle independence after surgery. Additionally, the light-energy distribution ought to be taken into account, particularly when choosing an IOL that favors the intermediate focus over the near one, as shown by the Triumf results (Figure 3). Although all the studied IOLs had superior far-focus in terms of the recorded optical quality at 3 mm, some IOL models can show deviations from this rule at lower and higher apertures. Therefore, assessing photopic and scotopic pupil size should further facilitate predicting postoperative visual quality. The broad range of SA reported in the general population makes it challenging to give a clinical interpretation of these laboratory results. The potential benefits of chromatic aberration in extending the depth of focus of pseudophakic eyes require further studies.

## Data Availability

Data is contained within the article.
